# Monitoring Cerebral Oxygenation During Neonatal Cardiac Surgery: Limitations of Conventional NIRS

**DOI:** 10.1002/pan.70183

**Published:** 2026-04-10

**Authors:** Nicolina R. Ranieri, Rodrigo M. Forti, Wesley B. Baker, Susan C. Nicolson, Jennifer M. Lynch

**Affiliations:** ^1^ Division of Cardiothoracic Anesthesiology Children‘s Hospital of Philadelphia Philadelphia Pennsylvania USA; ^2^ School of Biomedical Engineering, Science and Health Systems Drexel University Philadelphia Pennsylvania USA; ^3^ Division of Neurology Children’s Hospital of Philadelphia Philadelphia Pennsylvania USA; ^4^ Department of Neurology, Perelman School of Medicine University of Pennsylvania Philadelphia Pennsylvania USA; ^5^ Department of Anesthesiology and Critical Care, Perelman School of Medicine University of Pennsylvania Philadelphia Pennsylvania USA; ^6^ Department of Bioengineering, School of Engineering and Applied Science University of Pennsylvania Philadelphia Pennsylvania USA

1

Congenital heart disease (CHD) is the most commonly diagnosed congenital disorder, and approximately one‐third of affected children have critical CHD requiring surgical intervention in the neonatal period [1]. Improved survival of children born with critical CHD over the last few decades has highlighted the increased risk for neurodevelopmental disability in these patients. Although this risk is multifactorial, intraoperative risk factors such as use and duration of deep hypothermic circulatory arrest (DHCA), cooling duration and target temperature, and use and flow rate of regional cerebral perfusion have been linked to neurologic injury and poor outcomes [2, 3, 4]. Anesthesiologists rely on real‐time monitoring of cerebral oxygen saturation with near‐infrared spectroscopy (NIRS) to assess whether the brain is receiving adequate oxygen delivery during surgery. Failure to detect insufficient oxygen delivery impairs the ability to develop clinical protocols to mitigate this intraoperative risk.

Currently available clinical continuous‐wave near‐infrared spectroscopy (CW‐NIRS) devices emit constant‐intensity light on the skin and detect the intensities of the re‐emerging diffuse light exiting the tissue. CW‐NIRS devices, however, are limited by their inability to resolve tissue scattering from absorption and thus have typically only been used as relative trend monitors of regional oxygen saturation (rSO_2_). These devices have also been shown to be inaccurate even as trend monitors under certain physiologic conditions (e.g., during circulatory arrest) that alter tissue optical properties [4].

Several advanced NIRS approaches have been developed to address these limitations by estimating both tissue absorption and scattering optical properties. One example is time‐domain near‐infrared spectroscopy (TD‐NIRS), which measures the temporal distribution of photon flight times through tissue, allowing estimation of optical properties and quantitative assessment of tissue oxygenation. These techniques have been explored in pediatric cardiac surgery applications [5]. Another approach is frequency‐domain near‐infrared spectroscopy (FD‐NIRS), also referred to as frequency‐domain diffuse optical spectroscopy (FD‐DOS), which employs intensity‐modulated light that enables separation of the scattering and absorption optical properties of tissue, allowing for accurate quantification of absolute cerebral tissue oxygen saturation (ScO_2_) [6], as well as direct estimates of hemoglobin concentration. This capability allows FD‐DOS to provide a more precise measurement of cerebral physiology, potentially overcoming the limitations of trend‐based CW‐NIRS. In this report, we focus on FD‐DOS and illustrate its advantages compared to conventional CW‐NIRS during neonatal cardiac surgery.

Figure 1 showcases the advantage of FD‐DOS [4, 7] (Imagent, ISS Inc.) over CW‐NIRS (INVOS Model 5100c) on a representative neonate undergoing the Norwood procedure, which was partially performed under deep hypothermia and antegrade cerebral perfusion (ACP). For the FD‐DOS probe used in this report, source‐detector separations (SDS) ranged from 1.5 to 3 cm. Given that mean photon sampling depth is approximately half of SDS [6], this corresponds to an estimated sampling depth of ~0.75–1.5 cm below the skin, which is sufficient to measure the brain in neonates. FD‐DOS data were acquired and averaged in 10 s blocks and time synced with CW‐NIRS. For visualization purposes in Figure 1, a Gaussian smoothing filter was applied to both signals to improve clarity. This example illustrates a recurring pattern that was observed across multiple subjects, in which CW‐NIRS behavior diverges from expected physiological changes upon cooling to deep hypothermia. Notably, CW‐NIRS and FD‐DOS exhibited similar directional trends before and after cardiopulmonary bypass. However, once cardiopulmonary bypass and cooling initiated, rSO_2_ values from CW‐NIRS rapidly approached the device's upper limit, saturating at 95%, and remained essentially unchanged until rewarming. This saturation corresponds to the upper measurement limit of the INVOS device and limits sensitivity to ongoing physiological changes for much of the procedure. In contrast, FD‐DOS derived ScO_2_ remained dynamically responsive across all phases of the procedure, including a measurable decline in cerebral oxygenation during ACP that was not detected by CW‐NIRS.

**FIGURE 1:**
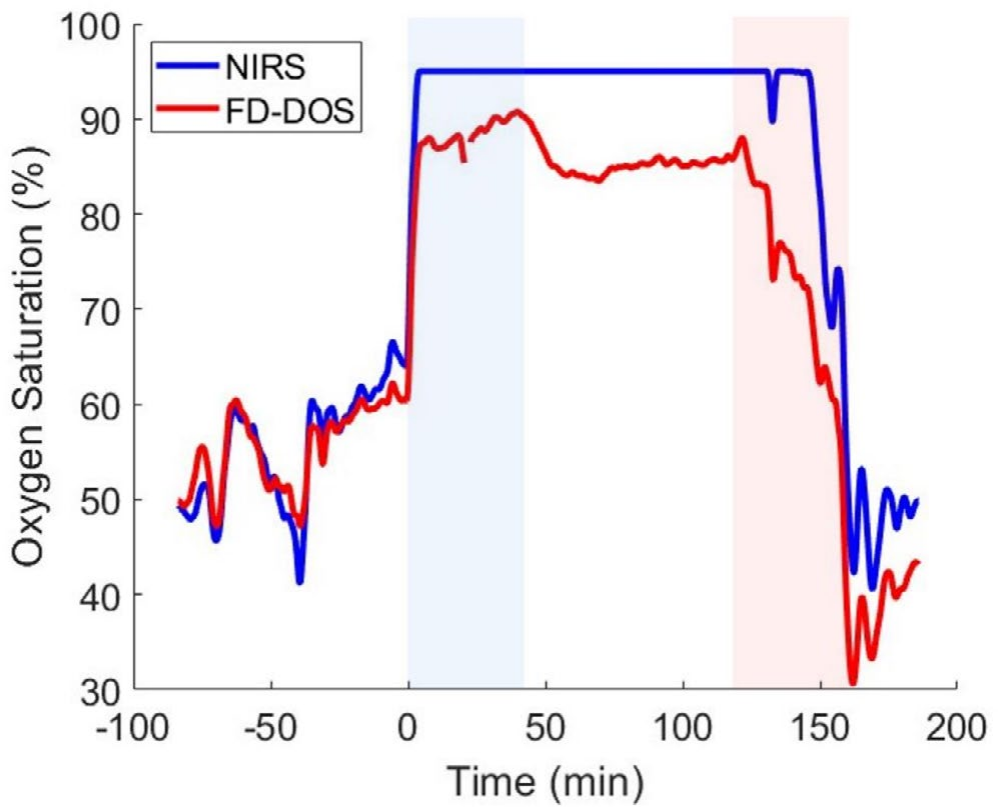
Comparison of cerebral oxygenation trends using FD‐DOS (ScO_2_, blue) and CW‐NIRS (rSO_2_, red) in a subject during aortic arch reconstruction with ACP. The cooling phase is shaded in blue, rewarming in red, and ACP is represented by the area between them. NIRS saturation at 95% during cooling, ACP, and rewarming fails to track oxygenation changes.

These limitations of CW‐NIRS demonstrated here are consistent with prior phantom and experimental studies demonstrating the tendency of CW‐NIRS devices to saturate under conditions of high oxygenation and hypothermia [4, 8]. Despite these known technical limitations, CW‐NIRS remains widely used for intraoperative neuromonitoring [9] and is increasingly relied upon in research applications. Research applications include the assessment of cerebral autoregulation during neonatal cardiac surgery; autoregulation indices rely on slow fluctuations in cerebral oxygenation. However, the use of CW‐NIRS signals that are prone to saturation during ACP can lead to inaccurate assessment of the slow fluctuations in ScO_2_, which can compromise physiological interpretation. Increased awareness of these limitations and consideration of advanced optical techniques such as FD‐DOS, may support more reliable assessment of cerebral oxygenation and cerebrovascular regulation.

In conclusion, differences in signal behavior observed between FD‐DOS and conventional CW‐NIRS show important limitations of CW‐NIRS during neonatal cardiopulmonary bypass under deep hypothermia. The reduced responsiveness of CW‐NIRS signals during procedures performed under hypothermia may limit physiological interpretability when accurate cerebral monitoring is most critical. Awareness of these constraints is important when using NIRS to guide intraoperative interpretation during complex neonatal cardiac surgery.

## Funding

This work was supported by CHD Coalition.

## Ethics Statement

This study was approved by the Institutional Review Board of Children's Hospital of Philadelphia (11‐008191, initially approved August 08, 2011). Parents were approached for written informed consent prenatally; for those not enrolled prior to delivery, consent was obtained postnatally.

## Conflicts of Interest

The authors disclose partial ownership of the following patents that are directly or indirectly related to FD‐DOS technology: WO2021/091961 (WBB), PCT/US2025/042798 (WBB+RMF). No author currently receives royalties or payments from these patents.

## Data Availability

The data that support the findings of this study are available from the corresponding author upon reasonable request.
